# Reappraising the Surgical Approach on the Perforated Gastroduodenal Ulcer: Should Gastric Resection Be Abandoned?

**DOI:** 10.4021/jocmr608w

**Published:** 2011-09-26

**Authors:** Kazuaki Kuwabara, Shinya Matsuda, Kiyohide Fushimi, Koichi B. Ishikawa, Hiromasa Horiguchi, Kenji Fujimori

**Affiliations:** a Kyushu University, Graduate School of Medical Sciences, Department of Health Care Administration and Management, 3-1-1 Maidashi, Higashi-ku, Fukuoka 812-8582, Japan; b University of Occupational and Environmental Health, Fukuoka, Japan; c Tokyo Medical and Dental University, Tokyo, Japan; d National Cancer Center, Tokyo, Japan; e The University of Tokyo Graduate School of Medicine, Tokyo, Japan; f Hokkaido University, Sapporo, Japan

## Abstract

**Background:**

Advancements in medical care for peptic ulcer disease (PUD) have reduced the need for invasive surgical procedures such as gastric resection (GR). Community-based PUD studies from a large sampling of PUD patients designed to analyze hospital resource use and outcomes after different surgical procedures have been rare. We aimed to exhaustively reappraise the risk factors and patient demographics that affect PUD patient recoveries after GR compared to those after simple closure (SC).

**Methods:**

We used a Japanese administrative database for 6 consecutive months each year between 2006 and 2010. The database included a total of 68,432 PUD patients; we analyzed 6,334 perforation cases and 3,148 cases of patients who underwent GR or SC. Study variables were demographics, comorbidities, characteristics of PUD, and operative day. Study outcomes that were analyzed included mortality, postoperative complications, ventilation administration, postoperative blood transfusions, length of stay, total charges, operating room (OR) time, and the postoperative fasting period (defined as the day of surgery to the day oral food intake was resumed.) To reduce selection bias in study procedures and to control the variation in hospital practice, a propensity score (PS) matching cohort analysis and a mixed linear regression model were used to assess the effects of GR on the outcomes.

**Results:**

In 699 hospitals, 322 GRs and 2,826 SCs were observed. Younger age, duodenal ulcers, preexisting anemia and an operative day no more than 24hours were significant associated with the choice of SCs. No significant differences were observed in study outcomes after either GR or SC; more postoperative blood transfusions and longer OR times but shorter postoperative fasting periods were observed after GR. Longer OR times, ventilation and postoperative blood transfusion were significantly associated with mortality. Not GR but longer OR times use of ventilation and complications were the most significant indicators of increased resource use.

**Conclusions:**

There were no major significant differences in GR when compared to SC with regards to patient recoveries. Surgeons should obtain the skills and establish strategies to optimize either type of surgical procedure including minimizing OR time and establishing the best perioperative critical care.

**Keywords:**

Peptic ulcer perforation; Simple closure; Gastric resection; Outcome; Resource use

## Introduction

Major improvements in medical treatment for peptic ulcer diseases (PUDs), including the introduction of H2 receptor antagonists, use of proton pump inhibitors, and *Helicobactor pylori* eradication with antibiotics, has significantly reduced the need for surgical treatment of the disease. However, the use of emergency surgery has recently increased in complicated PUD cases in which perforation or bleeding is present [1-3]. Use of laparoscopic techniques for simple closure (SC) of perforated PUDs has gradually replaced the open SCs [4-8].

Paimela et al reported that the trend for using local, simple surgical closures of PUDs in Finland has increased annually although the actual annual number of surgical procedures has decreased [1]. A shift from gastric resection (GR) or acid reducing surgery to local procedures such as SC has been observed. The use of gastrectomy with or without vagotomy was reported to be required in less than 10% of the cases; definitive surgery such as GR or vagotomy might have also become outdated [1,2]. An increase in aging populations in developed countries is occurring; these ageing patients are likely to have more comorbid conditions necessitating an increase in the use of non-steroid anti-inflammatory drugs. Patients with complicated PUDs in addition to other concomitant critical illnesses may have delayed recoveries even when innovative surgical techniques such as laparoscopic surgery are used. An increase in the number of emergency operations and no change in the number of suture repair on the perforated PUDs were also discussed [1,2].

In order to accommodate the changing trends in the demographics (primarily increased age and comorbidities) and a gradual decrease in surgical procedures, studies focusing on the use of SC procedures such as laparotomy or laparoscopy have been done. These studies identified risk factors associated with the simple surgical methods and information obtained from these studies have been and can be used to define conditions that can be used to control complications and decrease mortalities [9-14]. Age, procedure timing, concurrent ulcers and critical conditions such as sepsis were indicators of the type of surgical procedure or increase in mortality rate. Overall, effective perioperative management strategies were advocated based on evidence from these studies that were either single center studies or systematic reviews [5,6,11]. Generalizability, heterogeneity of patient-case-mix and the variations in treatment strategies were also expected to exist in study methodology or between hospitals. Advanced perioperative management would not necessarily eliminate the need for definitive or invasive GRs for complicated PUDs, nor would the use of simple, local procedures necessarily eliminate the recurrence of PUDs.

Using the Japanese administrative database containing a high volume of PUD surgical cases, we reappraised the use of GR versus SC techniques as part of the overall management strategy for patients with perforated PUD. In this study, we first examined the risk factors associated with SC methods, then constructed propensity score matching cohorts and finally investigated whether SC is a better surgical technique and leads to better patient recoveries than GR.

## Methods

### Study database

We utilized a Japanese administrative database established by the Ministry of Health, Labor and Welfare (MHWL) to develop an original Japanese case-mix classification and to determine the payment system. This database was established in Fiscal year (FY) 2002 by the Ministry of Health, Labor and Welfare (MHWL) and our research team. It was used to profile hospital performance and assess hospital payments from 1,428 hospitals (84 academic hospitals and 1,344 community hospitals) in 2010. The hospitals involved are responsible for delivering acute care, promoting medical research and educating students and postgraduate trainees. Among these hospitals, 1,030 hospitals voluntarily participated in our research project in order to refine the case-mix classification and to contribute information about medical care plans.

This national database contains discharge summaries and medical claims data for each hospital. Information on the number and dates of cases is collected annually between July 1 and December 31. We analyzed patients with perforated gastroduodenal ulcers who underwent surgical procedures at hospitals participating in this case-mix project. Our original database maintained by MHLW included a total of 9,320,681 patients across 1,067 hospitals from 2006 to 2010. Among those, 68,432 PUD patients from 1,030 hospitals were identified.

Our research project was approved by the Ethics Committee of the University of Occupational and Environmental Health (Fukuoka, Japan).

### Definitions of study variables and outcomes

The study variables were age, sex, use of an ambulance, principal diagnosis at admission (including the location of PUD) and the presence of concurrent bleeding, comorbidities including preexisting PUD, complications, type of PU surgical procedure, associated procedures during the primary surgery (use of laparoscopy and vagotomy), the actual day of surgery after admission (≤ 24 or > 48 hours), preoperative blood transfusions, the use of nasogastric tubes, ventilation administration, and hospital teaching status. Variables defining resource use were length of stay (LOS; days), total charges (TC; 1 Euro = 120 yen) and operating room (OR) time (min). TC was observed to be a good approximation for the in-hospital cost [15]; OR time was defined as the time required for the anesthesia procedures, preparation and positioning of video-imaging equipment, and surgical skin-to-skin time. Study outcomes analyzed were mortality, complications, use of ventilation, postoperative blood transfusion(s), LOS, TC, OR time and postoperative fasting period (defined as the time semi-solid food intake was resumed).

Age groups were categorized as follows: ≤ 39, 40 - 64 and ≥ 65 years. The diagnoses in the database were recorded in accordance with the International Statistical Classification of Diseases, 10th version (ICD10). The location of the perforated ulcer was categorized into stomach or duodenum as follows: stomach (K251-2, K255-6,); duodenal (K261-2, K265-6). Concurrent bleeding was considered to be present if their ICD code was K252, K256, K262 or K266.

Study surgical procedures were classified as GR including GR with or without reconstruction, or as SC with or without omental patch. Omental patch alone over the perforation was designated as SC. We also examined the concurrent use of vagotomy or laparoscopy. Conversion cases from laparoscopic approach to open traditional ones were counted as open cases. A maximum of four comorbidities per patient were recorded. To assess the severities of preexisting comorbidities, we used the Charlson comorbidity index (CCI) defined by ICD10 code [16]. CCI originally included PUDs and we considered the pre-existing PUD to be a comorbidity when the patients had an ICD10 code corresponding to PUD. A maximum of four unexpected complications during hospitalization were recorded separately in this database. Study complications were considered procedure-related complications classified as any of the following ICD10 codes (T81-T87) and surgical intra-abdominal complication of acute pancreatitis (K85), bowel obstruction (K560, K562, K565-7, K660 and K913), and of peritonitis/intra-abdominal abscess (K650 and K658-9) [17]. The database contains the dates of every medical care item and we calculated the amount of blood used for perioperative blood transfusions when required, postoperative fasting period (defined as the number of days after surgery that semi-solid food intake was resumed). Patients receiving preoperative blood transfusions were defined as those with pre-existing anemia. We divided hospital types into community and academic.

### Statistical analysis

Categorical variables were reported as the number and proportions of each of the different study surgical procedures used and compared using Pearson chi-square test. Continuous variables were compared between GR and SC using analysis of variance. To reduce the selection bias, the propensity score (PS) of providing SC was measured using a multiple logistic regression model incorporating the following covariates: the three age categories, sex, concurrent bleeding, location of PUD, CCI, the need for preoperative blood transfusion or pre-existing PUDs, indication for laparoscopy, operative period ≤ 24 hours, and hospital teaching status [18,19]. The calculated PS was used to designate paired matching cohorts for SC and GR. LOS and TC, OR time, blood transfusion, and the postoperative fasting period were compared for GR and SC. A multiple logistic regression model was used to determine the impacts of GR on study outcomes. A mixed linear regression model, where study hospitals were handled as random intercepts, was used to estimate the impact of GR on study resource use. We used the statistical analysis of SPSS 16.0. All reported p values were two-tailed, and the level of significance was set at p < 0.05.

## Results

Out of 6,334 perforated PUD patients, 3,148 patients in 699 hospitals were treated with study surgical procedures. There were 322 patients undergoing GR in 215 hospitals and 2,826 SC patients in 667 hospitals (GRs were done in 203 community and 12 academic hospitals; SCs were done in 614 community and 53 academic hospitals).

**Figure 1:**
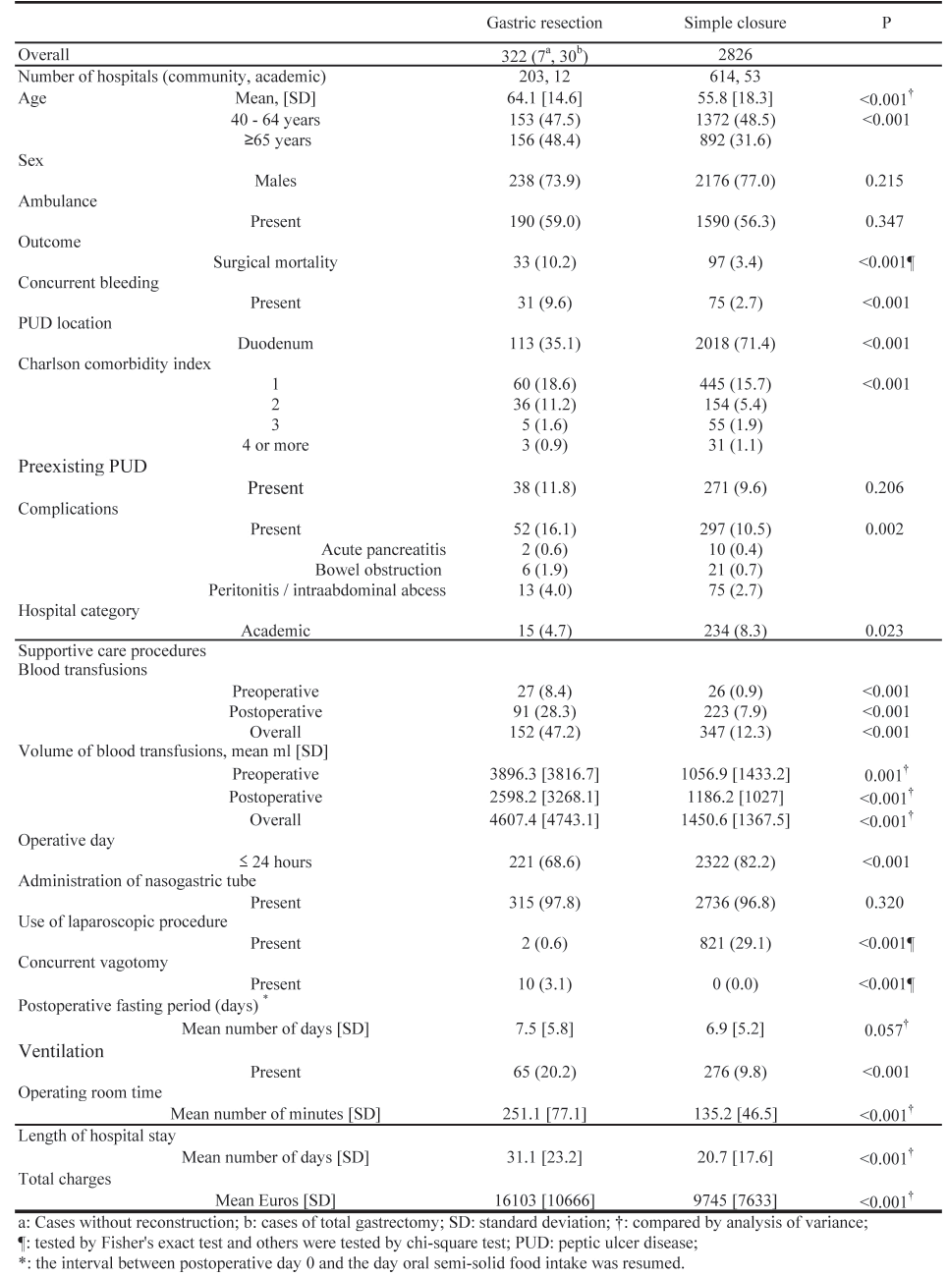
Patient Characteristics, Care procedures and Resource Use for Gastric Resection and Simple Closure.

The mean patient ages differed significantly between GR and SC (GR: 64.1 years versus SC: 55.8 years), and the proportion of patients aged 65 years who underwent GRs was greater (48.4%) than those who underwent SCs (31.6%). Higher frequencies of pre-existing comorbidities, complications, mortality, use and amount of blood transfusions, and ventilation administration were observed in GRs, whereas frequent duodenal perforation, shorter LOS or OR time and less TC were seen in SCs. However, postoperative fasting periods were not significantly different between GRs and SCs (Fig. 1).

**Figure 2:**
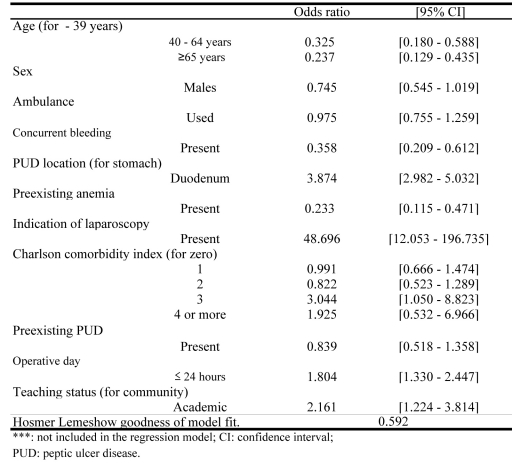
Variables Associated With Indications for Simple Closure.

Figure 2 indicates the study variables associated with the indications for SC. Advanced age, duodenal ulcer, pre-existing anemia and operative period over 24 hours significantly correlated with SC [Odds ratio (OR) 95% confidence interval (95%CI)]: 40 - 64 years 0.325 (0.180 - 0.588); no less than 65 years 0.237 (0.129 - 0.435); duodenum 3.874 (2.982 - 5.032); preexisting anemia 0.233 (0.115 - 0.471); indication of laparoscopy 48.696 (12.053 - 196.735); operative day no more than 24 hours 1.804 (1.330 - 2.447) (Fig. 2).

**Figure 3:**
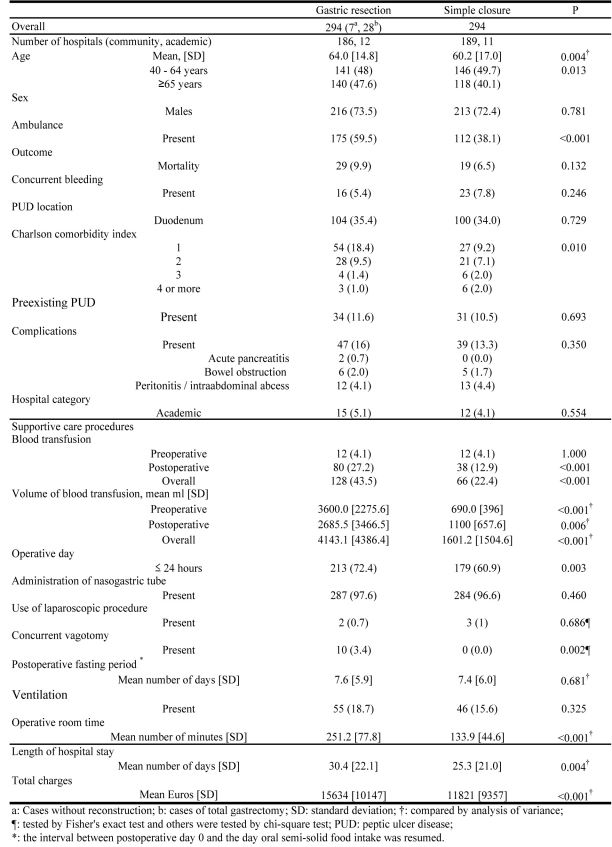
Patient Characteristics, Care Procedures and Resource Use for Gastric Resection and Simple Closure After Propensity Score Matching.

From the PS matching cohort analysis of GR and SC, shorter LOS and OR time and less TC were observed in SCs compared with GRs. There was no difference in ventilation administration and in postoperative fasting periods (Fig. 3).

**Figure 4:**
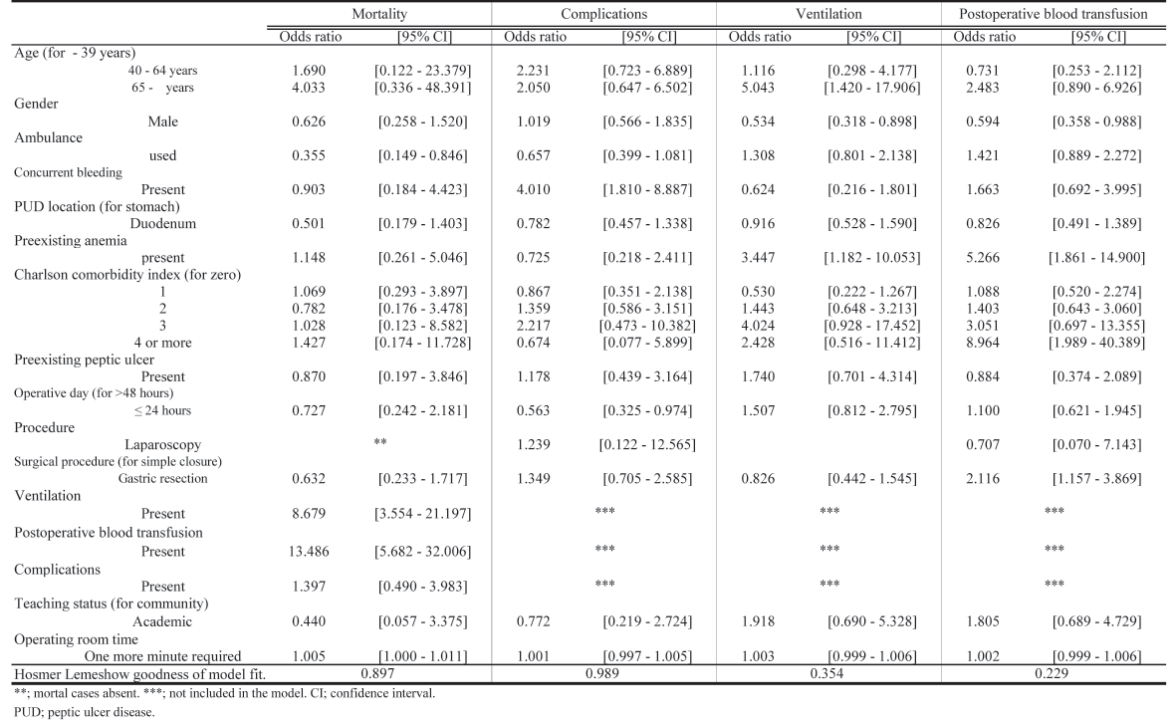
Variables Associated with Mortality, Complications, Ventilation and Postoperative Blood Transfusion.

GR was a significant indicator for postoperative blood transfusions except in cases of mortality, those with complications and ventilation [OR (95%CI): 2.116 (1.157 - 3.869)]. The need for ventilation and blood transfusions and prolonged OR time correlated with an increase in mortality [OR (95%CI): 8.679 (3.554 - 21.197), 13.486 (5.682 - 32.006), 1.005 (1.000 - 1.011), respectively] (Fig. 4).

**Figure 5:**
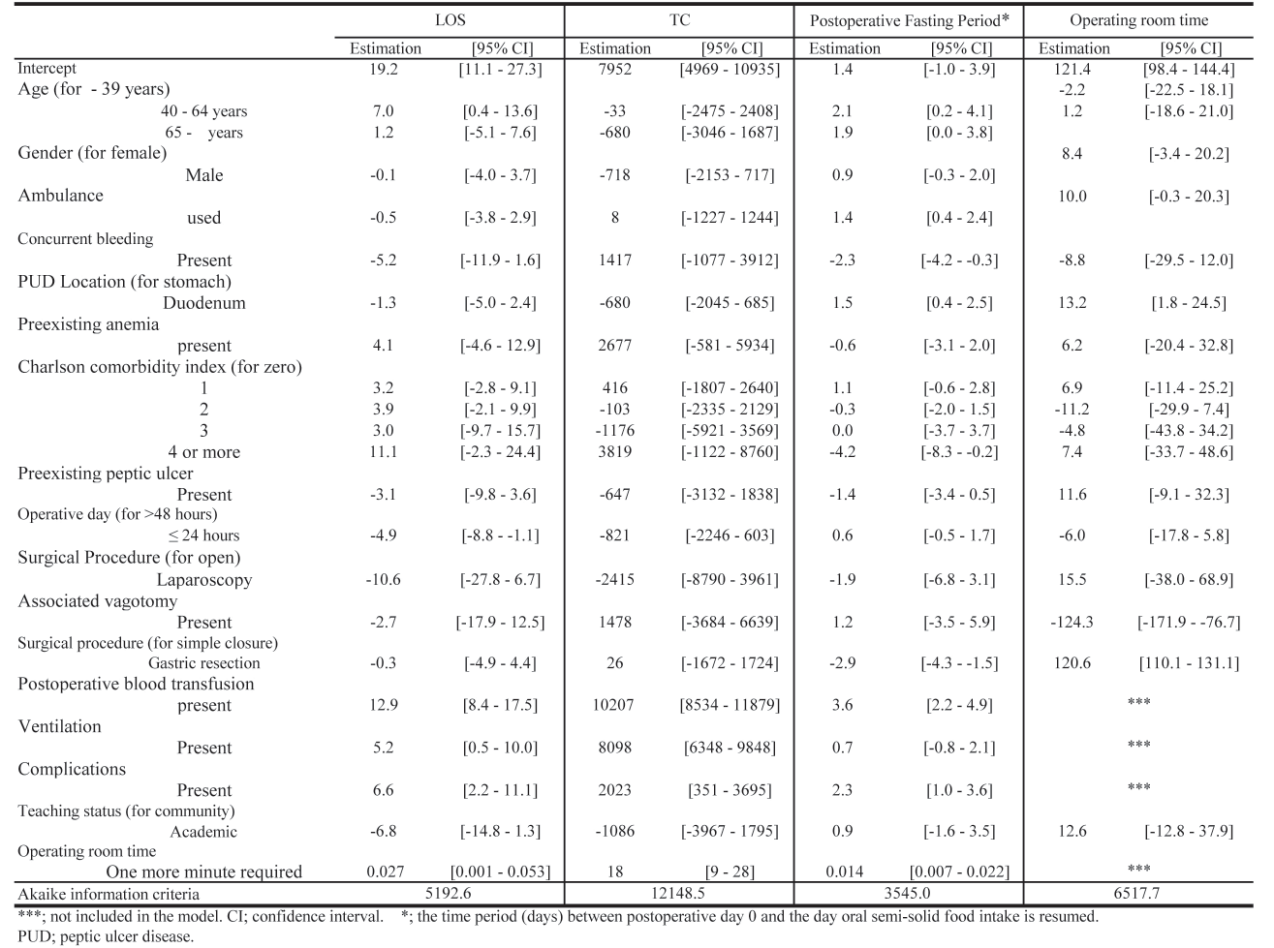
Variables Associated with Length of Stay (LOS), Total Charge (TC), Operating Room Time (min) and Postoperative Fasting Period.

GR determined shorter postoperative fasting periods and longer OR times, postoperative fasting period (days): -2.9 (-4.3 鈥?-1.5); OR time: 120.6 (110.1 - 131.1), but there was not a significant difference in LOS and TC between GR and SC. Longer OR time extended the postoperative fasting period significantly, 0.014 (0.007 - 0.022) (Fig. 5).

## Discussion

Using the Japanese administrative database, we analyzed the different surgical procedures with respect to mortality, postoperative complications and the need for ventilation and postoperative blood transfusions, LOS, TC, postoperative fasting periods, and OR time. To date, this quantitative study has the largest sample size of GR or SC cases for perforated PUDs from community-based hospitals. Using PS pair matching analysis and by controlling the selection bias, this study demonstrated that GR was not always less advantageous in terms of resource use or on the overall outcome. GR only determined the postoperative blood transfusions and longer OR times (that significantly delayed postoperative resumption of semi-solid food intake), although GR significantly accelerated postoperative oral intake. Longer OR times might deter the recovery of bowel motility, and physician’s concern about omental patch alone over the larger sized perforation, categorized as ‘SC’ in this study, might halt his decision on resuming postoperative oral intake.

Perforated PUD, whether treated either by GR or SC, may be complicated by perioperative factors associated with various critical conditions that may require respiratory care, or by complications in which there is a delay in the return to normal bowel motility due to prolonged bowel paralysis as a result of intra-abdominal infections [14]. It should be cautioned that GR was found to significantly correlate with the requirement for postoperative blood transfusions, and in some cases led to mortality. However, these findings indicate that in general the perioperative management of complicated conditions rather than the actual surgical procedures is crucial to patient recovery. Advanced age, cardio-pulmonary comorbidities, elevated shock status [as defined by the American Society of Anesthesiologists (ASA) or Boey scores], and septic conditions caused by a preoperative delay of over 24 hours have often been defined as risk factors for complications or mortality [5, 9-11]. Ventilation administration was an indicator of higher mortality in our study; these results correlate with results from previous studies.

The recent development and implementation of international guidelines for management of severe sepsis and septic shock or multidisciplinary care in the intensive care unit (ICU) might decrease the incidence of mortality that was observed in complicated PUDs until 2004 [20,21]. The relatively low number of gastric procedures performed in western countries, compared with Japan, might not enable western clinicians to undertake a community-based study such as ours [22]. The proportion of perforation was 9.3% in our study, which corresponded to the findings of Wang et al in which they reported a 9.2% perforation rate in 2006 in the United States [2]. However, the mortality rate in our study was 10% in cases of GR and 3.4% in cases of SC; this was significantly lower than that in the previous report by Wang et al, in which surgical mortality was 15% from 1993 to 2006 [2]. The trend towards lower mortality was also seen in gastric cancer, where the proportion of mortality was approximately 1% and 6% in Japan and western countries, respectively [22,23]. In this aging population it is expected that there will be increasing numbers of PUD patients and the surgical procedures for complicated PUDs will need to be improved to deal with the increasing number of cases. Comparative quality or risk identification studies should continue to be updated, not limited to PUD cases, but for any type of surgical procedure. This suggestion is validated by our findings that a longer OR time was found to be another risk factor for mortality, more resource use and also delayed the return to postoperative oral food intake. Surgeons should gain or maintain the skills to shorten the OR time in addition to identifying the high risk patients before selecting the type of surgical approach. Community-based appraisals should continue to be updated according to the development of perioperative care strategies and the changing trends in patient demographics.

Some limitations of this study should be discussed. First, this study was observational and obtained from discharged patients, so that we did not control study variables. Information was gathered over consecutive 6 month periods (from July 1 to December 31) over four years (2006 - 2010). However, the MHLW has included all of the hospitals participating in this case-mix project and extended the study period to 12 months as of 2010. Our study database included a sufficient sample size compared with other study databases so that our findings will not change significantly even if new data are added.

Second, we did not include an analysis of some important clinical data such as the precise onset time of perforation, perforation size, severity score (based on the Boey or ASA scores that are often used in PUD studies) and the types of SC technique. To predict the onset time of the perforation as precisely as possible, an electronically formatted database with outpatient cases will be required to be linked to the MHLW database. ASA scores were gathered in this study but the percentage of missing ASA scores in PUD cases (84.5%) was significant. There have been some critiques that the Boey or ASA scores are too subjective. A more objective indicator such as volume of required preoperative fluid that might be more representative of the shock status defined by the Boey score, may be more useful and feasible in future studies using this administrative database [10,14]. SC with or without omental patch and omental patch alone over the perforation were summarized into the same surgical procedure code in Japanese claim schedule. The sophistication of surgical procedure codes is underway in cooperation of MHLW and Japanese Society of Surgery.

Third, the LOS for all hospital admissions in Japan is three to four times longer than that in hospitals in Western countries [24]. One reason for the longer LOS is that Japanese hospitals generally supply a nursing service in addition to acute medical care [25]. However, the fiscal impact of longer LOS is reflected in the total costs for each case study.

In conclusion, using an administrative database and PS pair matching analysis, we quantitatively reappraised the quality of GR and SC for perforated gastroduodenal ulcers. There were no significant differences in mortality, complications, need for ventilation and resource use between SC and GR. However, GR consumed more OR time and required more postoperative blood transfusions. Ventilation, complications and longer OR times were associated with an increase in mortality and resource use. Surgeons should attain the surgical skills to reduce OR times rather than the emphasis being on the type of surgical procedure, it could then be on perioperative care strategies.
